# Planning training seminars in palliative care: a cross-sectional survey on the preferences of general practitioners and nurses in Austria

**DOI:** 10.1186/1472-6920-10-43

**Published:** 2010-06-11

**Authors:** Gerhild Becker, Felix Momm, Peter Deibert, Carola Xander, Annemarie Gigl, Brigitte Wagner, Johann Baumgartner

**Affiliations:** 1 Department of Internal Medicine II, University Medical Center Freiburg, Freiburg, Germany; 2 Palliative Care Research Group, University Medical Center Freiburg, Freiburg, Germany; 3 Red Cross Austria, National Association Styria, Graz, Austria; 4 External consultantions for Socioscientific Studies, Methodology and Statistics Graz, Austria; 5 Palliative Care Coordination Styria, KAGes-Services Graz, Austria

## Abstract

**Background:**

Training in palliative care is frequently requested by health care professionals. However, little is known in detail about the subject matters and the educational preferences of physicians and staff or assistant nurses in this field.

**Methods:**

All 897 registered GPs and all 933 registered home care nurses in the district of Steiermark/Austria were sent postal questionnaires.

**Results:**

Results from 546 (30%) respondents revealed that GPs prefer evening courses and weekend seminars, whereas staff and assistant nurses prefer one-day courses. Multidisciplinary sessions are preferred by almost 80% of all professional groups. GPs preferred multi disciplinary groups most frequently when *addressing psychosocial needs *(88.8%) and *ethical questions *(85.8%). Staff and assistant nurses preferred multidisciplinary groups most frequently in the area of *pain *management (88%) and opted for multi disciplinary learning to a significantly higher extent than GPs (69%; p < 0.01). Those topics were ranked first which are not only deepening, but supplementing the professional training. On average, GPs were willing to spend a maximum amount of € 400 per year for training seminars in palliative care, whereas nurses would spend approximately € 190 for such classes.

The results provide a detailed analysis of the preferences of GPs and nurses and offer guidance for the organisation of training seminars in palliative care.

**Conclusions:**

Medical and nursing education programs often pursue separate paths. Yet our findings indicate that in palliative care multidisciplinary training seminars are favoured by both, doctors and nurses. Also, both groups prefer topics that are not only deepening, but supplementing their professional knowledge.

## Background

For most European countries - except for the United Kingdom, where palliative medicine has been a medical speciality since 1987 [1], palliative care is a relatively new and developing medical speciality. In Austria, the hospice and palliative care movement emerged comparatively late in the early nineties of the last century, but now it shows a rapid development. In recent years, Austria has established a comprehensive palliative and hospice care service concept which is well integrated in the Austrian health care system. By implication, there is an increasing awareness on education and training in palliative care and a high need for undergraduate and postgraduate education and advanced training seminars for health care professionals. In addition, a fundamental training in palliative care should be integrated in the education of all medical professionals [2,3].

But education and training in palliative care still faces a number of significant difficulties. Although there have been considerable advances in *pain management *and *symptom control*, palliative care is under represented in medical education [4,5]. General practitioners and community nurses still feel insufficiently trained when they start to work in the field of palliative care [6-8]. Palliative care is important in general practice because the final year of a patients' life is usually spent at home under the care of a general practitioner (GP) and a primary health care team [9-11]. Research indicated that training in *symptom control, communication, counselling *and bereavement support is frequently requested by GPs and nurses [12-14]. Numerous reports document inadequate knowledge and education of healthcare professionals in *symptom management *and other palliative care skills [15-18].

Although a gradual expansion of palliative care education for health care professionals has taken place over the last few years, recent reports have shown that even in countries with a long lasting 'palliative care tradition' like the UK, efforts to further improve the education of health care professionals in palliative care are still falling short. [19]. The effectiveness of different methods of continuing professional education of GPs has been examined [20-22]. Research shows that traditional forms of education may not be effective in delivering positive changes in clinical behaviour; case-based education seems to be more effective [23-25]. To meet the interests and the needs of health care professionals in the field of primary palliative care, more information is needed on what kind of training in palliative care is required. Little is known in detail about the preferences of GPs and nurses regarding the specific design of training seminars in palliative care.

Against the background of the favourable development of palliative care in Austria we undertook this survey to identify the preferences of the GPs' and nurses' regarding the specific design of training seminars in palliative care. We wanted to gain a better insight into which educational topics, timeframe, location and group designs are likely to attract a majority of different professional groups. In addition, we are convinced that similar questions concerning the education in palliative care will arise in other countries in continental or eastern Europe which are currently working on the development of palliative and hospice care concepts.

## Methods

Structured postal questionnaires were sent to all 897 registered GPs and to all 933 registered home care nurses in the district of Steiermark/Austria. Overall, 1830 questionnaires were sent out. After a waiting period of 3 weeks, the database was closed and we started the data entries and the analysis. The questionnaires were developed by drawing on literature research [7,8,26,27]; they were pre-tested by GPs and nurses for face and content validity. Four-point Likert scales were used. In accordance with their job descriptions, questionnaires for GPs differed from those of the nurses. To collect information on the doctors' and nurses' preferences regarding the design of training seminars in palliative care, detailed questions were developed to gather more information on their preferred topics, time-frame, location, group design and own contribution to fees. Respondents also provided personal data and listed palliative care training sessions which they had attended in the last two years before the survey. A distinction was made between staff nurses and assistant nurses.

### Statistics

The comparison of frequencies was carried out by chi-square testing. Linear coherence was analysed by using the Spearman coefficient. To analyse differences between central tendencies, the Kruskal-Wallis-H-Test and Mann-Whitney-U-Test were used, which substituted parametric variance-analyses. P-values were corrected according to Bonferoni. The two-sided testing of hypotheses was carried out at a significance level of α = 0.05.

### Ethical approval

The study was approved by the Ethics Committee of the University Medical Center Freiburg.

## Results

The overall response rate to the questionnaire was 30% (546/1830), 25% of the GPs (228/897) and 34% (318/933) of the nursing staff members responded. The demographic characteristics of the respondents are shown in table 1.

**Table 1:** Demographic characteristics

	GPs	Staff nurses	Ass. nurses	Total sample
**Gender n (%)**				
Female	48 (22.4)	217(99.1)	82 (91.1)	347 (66.3)
Male	166 (77.6)	2(0.9)	8(8.9)	176 (33.7)
**Age [years]**				
Mean (range)	47.1 (26-77)	36.9 (20-55)	36.1 (19-59)	41.1 (19-77)
**Professional Life [years]**				
Mean (range)	18.8 (1-52)	12.5 (0.5-31)	5.6 (0.25-20)	13.9 (0.25-52)
				
**Trainings attended n (%)**				
Care for the dying	45 (22.7)	88(41.7)	36(43.9)	169 (32.3)
Palliative Care	87 (44.2)	89 (42.2)	29 (35.4)	205 (39.2)
Caregiver support	36 (18.3)	34(16.1)	23(28.0)	93 (17.8)
Psychological care	51 (26.0)	25(11.8)	22(26.8)	98 (18.7)
Pain management	140 (71.1%)	87 (41.2%)	16 (19.5%)	243 (46.5)
Overall	154 (78.6)	151 (71.6)	56(68.3)	361 (69.0)

**Figure 1:**
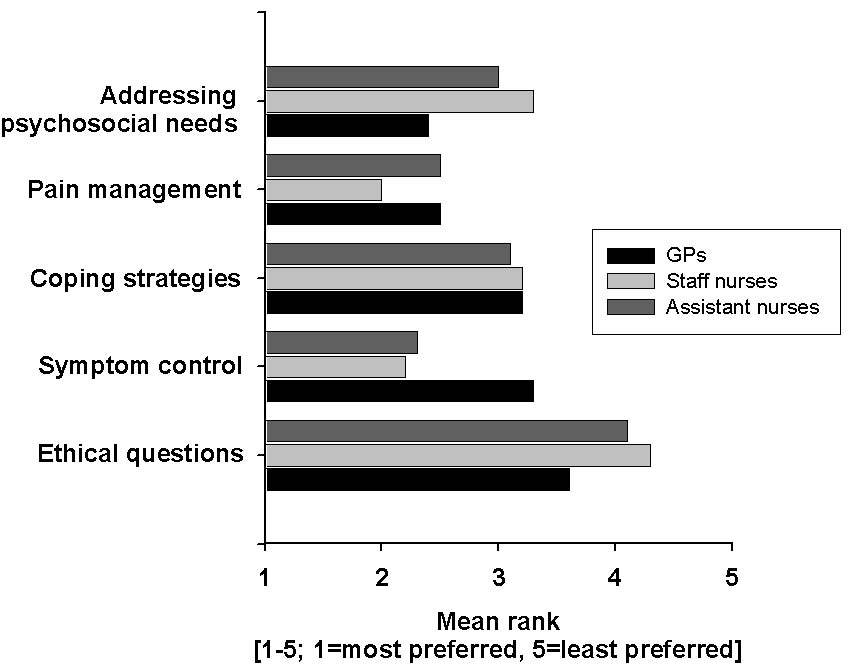
**Ranking of topics for training seminars in palliative care**.

When asked about their priority topics for training seminars in palliative care, the respondents ranked *pain management *first (mean rank 2.3). Detailed results about preferred seminar topics grouped by profession are depicted in figure 1.

**Figure 2:**
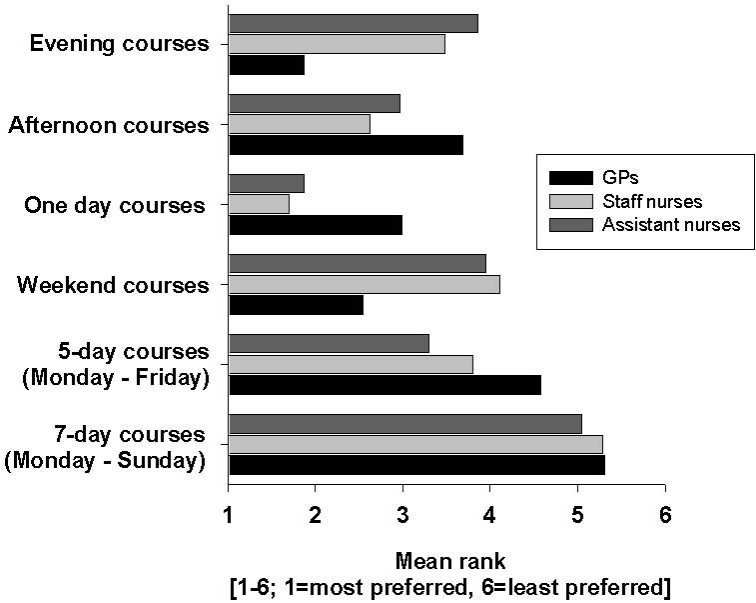
Preferred time frames for training seminars in palliative care; Significance *: p < 0.01.

The respondents were asked to rank six different time-frames for training courses in palliative care (figure 2). General practitioners preferred evening courses and weekend courses, whereas nurses preferred afternoon and one-day courses. All groups ranked 7-day courses (Monday - Sunday) lowest.

**Figure 3:**
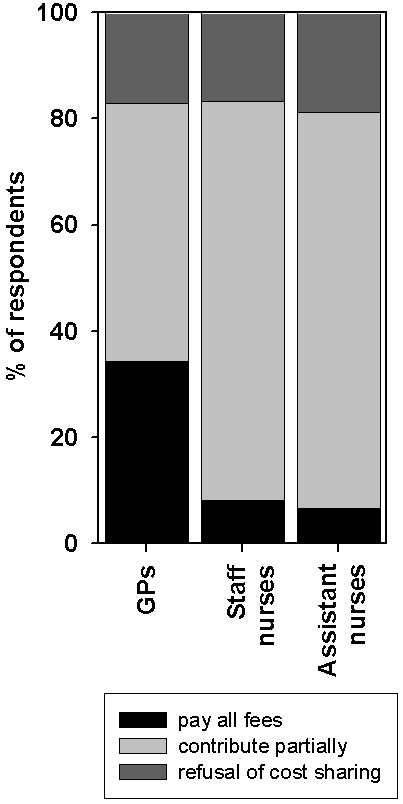
Training seminars in palliative care: readiness to contribute to fees; Significance *: p < 0.01.

18.9% (102/539 respondents) agreed to pay all fees for training seminars completely by themselves, 63.8% (344 respondents) agreed to contribute partially and 17.3% (93 respondents) refused any cost sharing. Specified results of the three professional groups are shown in figure 3. The majority of respondents would be prepared to spend a maximum amount of € 750 for palliative care trainings per year. The analysis on the professional groups showed that the most favoured amount that GPs are willing to pay on average is about € 410, staff and assistant nurses would favour to spend an amount of approximately € 190 (significant difference: p < 0.01) for palliative care trainings per year. Neither the span of professional life nor the self-estimated educational need in palliative care influenced these results.

Staff and assistant nurses were also asked about their willingness to attend training seminars during their leisure time. 52.7% of the staff nurses and 51.7% of the assistant nurses were disposed to do so.

89.7% of respondents agreed to take part in training seminars in their home region and 76.8% agreed to attend training seminars anywhere in the district. Statistically, there were no significant differences between the professional groups. 42.2% of all respondents agreed to attend seminars anywhere in their home country. 20.2% agreed to attend seminars abroad. General practitioners were significantly more often disposed to travel for seminars than nurses (p < 0.05).

Respondents were also asked if they preferred courses with members of their own occupational group only or whether they favoured multidisciplinary training courses. Overall assessment shows that multidisciplinary seminars are preferred by all professional groups irrespective of the topics.

**Figure 4:**
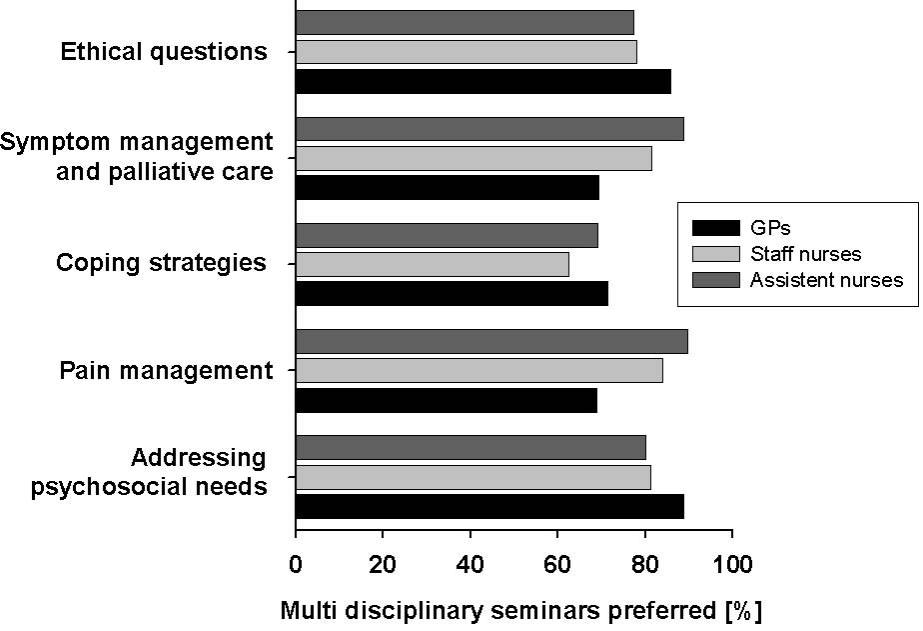
Preferred group design for seminars; Significance: *: p < 0.01.

Detailed analysis on the different topics is given in figure 4. GPs preferred multidisciplinary groups most frequently in *addressing psychosocial needs *(88.8%) and in *ethical questions *(85.8%). Staff and assistant nurses preferred multi disciplinary groups most frequently in the area of *pain managemen*t and opted for multidisciplinary learning significantly more often than GPs (p < 0.01).

Overall assessment showed that nearly 70% of all respondents had attended at least one training seminar in the last two years before the survey was conducted (Tab. 1). There was no statistically significant difference between the three occupational groups. Training courses in *pain management *were mostly attended. The analysis on the different professional groups and their years of professional life showed that no significant differences existed in the time spent in training seminars in palliative care.

## Discussion

### Educational topics

Palliative care issues seem to be of increasing importance in the field of daily medical practice. The majority of the health professionals doing our survey had attended at least one training seminar on palliative care topics in the last two years before the survey was conducted (69%, cf. table 1).

Regarding the ranking of priority topics in palliative care, all professional groups considered *pain management *and *symptom control *to be of prime importance, whereas *ethical questions *was considered to be the least relevant (figure 1).

GPs chose *addressing psychosocial needs of patients and their families *as the subject of highest priority, whereas staff nurses and assistant nurses ranked *pain management *and *symptom control *to be the most important educational issue in palliative care. These results are quite interesting, insofar as GPs did not prefer typical medical topics, and staff or assistant nurses did not favour typical nursing topics. These findings are concordant with previous smaller studies. Wakefield et al. found that the management of symptoms was an area of concern for many Australian GPs whereas *pain management *and the *management of patients' and carers' psychological distress *were not [27]. Trollor reported that Australian rural GPs felt competent to manage physical symptoms, but considered the psychological manifestations of palliative care very hard to cope with [28]. These findings were corroborated in American family physicians [29]. A Spanish study investigating the educational needs of primary health care professionals regarding the management of terminal cancer patients showed that doctors rated a training session in the area of emotional support higher than improving their handling of symptoms [30]. On the other side, a questionnaire survey in the UK found that community nurses rated pain control education a major issue [7]. These findings indicate the importance of choosing topics that are not only deepening, but supplementing professional education in advanced training sessions in palliative care.

In comparison to other fields in medicine, palliative medicine is a discipline which involves many emotional issues. In addition to physical difficulties, psychological, social and spiritual sufferings need to be assessed and treated. Although palliative care physicians are constantly dealing with sensitive and straining issues, surveys and self-ratings have highlighted deficiencies in end-of-life care in postgraduate medical training [31-33]. According to international reports [19,34-39], doctors and nurses appear to be inadequately trained in the field of palliative care which poses a serious problem for several countries.

### Timeframe and group design

Regarding the planning of future training seminars, our findings indicate that block seminars are not favoured, particularly when they take place longer than one week (figure 2). One-day or afternoon courses are most preferred by nurses, whereas GPs prefer evening and weekend courses. This is concordant with the findings of a questionnaire survey by Samaroo who found that nurses preferred one day-courses for training seminars in palliative care, whereas GPs preferred half day courses [8].

GPs are willing to pay significantly higher amounts for their further education than other professional groups; e.g. they would pay the complete fees for training seminars. GPs were also significantly more often disposed to attend seminars abroad than staff or assistant nurses. This is probably due to the fact that the average income of GPs is higher. An additional explanation might be that still the nurses are mainly female and that possibly women still might be involved in family obligations to a higher extent than their male colleagues.

Concerning the design of the educational settings, GPs as well as nurses prefer multidisciplinary groups in training seminars for palliative care. A multidisciplinary approach and participants from different disciplines seem to be important; especially for the teaching of psychosocial and ethical topics because these are important to all professional groups. But about 75% of the nurses and two-thirds of the GPs favour multidisciplinary group designs even in seminars on issues which are quite group-specific, for example issues like *symptom management *for GPs or *special palliative care techniques *for nurses.

Concerning seminars on *pain management*, our results show that nurses favour significantly more often a multidisciplinary group design than GPs.

In reality, medical and nursing education programs still often pursue separate paths.

Inter-professional relationships may be difficult due to the different philosophic approaches of care and other constraints imposed by time and funding imperatives [40]. Nevertheless, specialist palliative care depends particularly on effective multidisciplinary teamwork. Our findings indicate that in the field of palliative care multidisciplinary training seminars are favoured by both, doctors and nurses. Curriculum planners should be aware of these preferences.

### Strengths and limitations of the study

Due to the cost of our survey, no reminder was sent and the questionnaire was comparatively long (Please find the translated questionnaires as additional file 1 and additional file 2). Consistent with literature [41-43] we suppose that these factors diminished the response rate and thus may have introduced a non-respondent bias. It is possible that the samples consist mainly of GPs and nurses with the greatest interest in palliative care. However, age, gender and the duration of the professional activity of the respondents in this survey are similar to that of the practicing GPs and nurses in Austria in 2003-2004 [44]. Therefore, in spite of the low response rate, results might be judged as representative and permit inferences from the study sample to the whole population. Our survey, involving three different occupational groups, allows a detailed comparison of GPs, staff and assistant nurses and helps to plan future training seminars in palliative care. Results provide a detailed understanding of the needs and preferences and may help target goals for better organisation of training seminars in palliative care.

## Conclusion

Training in palliative care is frequently requested by health professionals. Medical and nursing education programs often pursue separate paths, but our findings indicate that in palliative care multidisciplinary training seminars are favoured by both, doctors and nurses. Topics which are not deepening, but supplementing professional training are required and wished-for by GPs and nurses. One-day or afternoon courses are preferred by nurses, whereas GPs prefer evening or weekend courses. The maximum amount that health professionals are willing to pay for depends upon the profession: GPS would pay approxiamtely € 400 per year and nurses would pay € 190. A suggestion for further research is to conduct longitudinal follow-ups in which the long-term effect of health professionals who are properly trained in palliative care programs is evaluated.

### Additional reference

We undertook this survey in order to get better insights how GPs' and nurses' themselves rate the competency and educational needs of their own professional group in palliative care and to target goals for training courses in palliative care. Therefore the questionnaire was comparatively long covering 16 different domains and asking a total of 76 items. Altogether 1830 persons were polled. In this way, we collected an amount of data. We decided to write two different papers both based on this large survey in order to present the results in a clear and manageable fashion. In our first publication [45] we investigated if there is a correlation between factors like professional education, years of professional life, number of attended specialised training sessions on the one hand and the self assessment of competency and educational needs on the other hand and if there are significant differences between the different professional groups. In the present paper we want to offer some guidance for the concrete organisation of training seminars in palliative care.

## Competing interests

The authors declare that they have no competing interests.

## Authors' contributions

GB participated in the study design, interpretation of data and drafted the manuscript. FM made substantial contributions to statistical analysis and helped draft the manuscript. PD made substantial contributions to the interpretation of the data and critically revised the manuscript. CX made substantial contributions to the interpretation of the data, she participated in drafting and revising the manuscript. AG participated in the study design, in the acquisition and interpretation of data and she contributed to the revision of the manuscript. BW performed statistical analysis, made substantial contributions to the interpretation of the data and to the revision of the manuscript. JB developed the idea and the design of the study, participated in the acquisition and interpretation of the data and in revising the manuscript.

## Pre-publication history

The pre-publication history for this paper can be accessed here:

http://www.biomedcentral.com/1472-6920/10/43/prepub

## Supplementary Material

Additional file 1 **Questionnaire GPs**. Original questionnaire for the GPs (translation into English) Click here for file

Additional file 2 **Questionnaire Nurses**. Original questionnaire for the nurses (translation into English) Click here for file
