# Supplementary material for: Planning training seminars in palliative care: a cross-sectional survey on the preferences of general practitioners and nurses in Austria
Source: BMC Med Educ. 2010 Jun 11;10:43. doi: 10.1186/1472-6920-10-43 (PMC2893516; doi:10.1186/1472-6920-10-43)
Supplement: Additional file 1 — Questionnaire GPs. Original questionnaire for the GPs (translation into English) [file 1472-6920-10-43-S1.PDF]

Questionnaire: mobile palliative care – GPs

Graz, September 2000

Dear Colleagues,

The care of critically ill or end-of-life patients has lately become an issue of growing interest. Internationally and in the Steiermark, hospitals are now planning to implement a graded care system for palliative care patients.

The goal of the questionnaire is to learn more about the specific academic requirements and the need for accompanying measures of the occupational groups who work in nursing care or end-of-life care.

Your opinion is very important to us! That is why we would ask you to fill out the whole questionnaire. You will need approximately 15-20 minutes.

Please answer every question!

Your answers will be kept strictly confidential towards third parties. The results of this questionnaire will be online in spring/summer 2001. In case you are interested, you will find them on the website of the FAGW: http://www.stmk.gv.at/gesundheit/.

Thank you very much for your time and your cooperation!

Dir. Annemarie GIGL　　　　Dr. Johann BAUMGARTNER

Questionnaire – general practitioners
Academic requirements for mobile palliative care

In the following pages, you will find questions which will deal with your occupation. Please tick the boxes which apply to you (if not indicated otherwise). Please answer every question!

Personal information

Age: …… years Sex: ☐ male ☐ female

Work experience:......… years

Completed degrees:

☐ Psycho-social medicine
☐ Psycho-somatic medicine
☐ Psycho-therapeutic medicine
☐ Geriatrics

Additional degrees ………………………………………………………

1. Please assess your occupational group's competence concerning the psychosocial care for critically ill or end-of-life patients and their relatives in the following points:

1.1. Communication with patients

☐	☐	☐	☐
sufficient	predominantly sufficient	predominantly insufficient	insufficient

1.2. Communication with relatives

☐	☐	☐	☐
sufficient	predominantly sufficient	predominantly insufficient	insufficient

1.3. Handling of introductory conversations

☐	☐	☐	☐
sufficient	predominantly sufficient	predominantly insufficient	insufficient

1.4. Conducting difficult conversations (dying, death, mourning, "last things", …)

☐	☐	☐	☐
sufficient	predominantly sufficient	predominantly insufficient	insufficient

1.5. Care of patients in their last days (terminal phase)

☐	☐	☐	☐
sufficient	predominantly sufficient	predominantly insufficient	insufficient

1.6. Care of relatives in the patient's last days (terminal phase)

☐	☐	☐	☐
sufficient	predominantly sufficient	predominantly insufficient	insufficient

1.7. Care of relatives after the patient’s death

☐	☐	☐	☐
sufficient	predominantly sufficient	predominantly insufficient	insufficient

2. Please assess your occupational group’s <u>competence</u> in <u>coping with emotional and physical strains</u> in the following points:

2.1. Coping strategies for difficult situations

☐	☐	☐	☐
sufficient	predominantly sufficient	predominantly insufficient	insufficient

2.2. Coping with own pain

☐	☐	☐	☐
sufficient	predominantly sufficient	predominantly insufficient	insufficient

2.3. Coping with powerlessness

☐	☐	☐	☐
sufficient	predominantly sufficient	predominantly insufficient	insufficient

2.4. Coping with insecurities

☐	☐	☐	☐
sufficient	predominantly sufficient	predominantly insufficient	insufficient

2.5. Coping with guilty conscience

☐	☐	☐	☐
sufficient	predominantly sufficient	predominantly insufficient	insufficient

3. Please assess your occupational group’s <u>competence</u> in <u>the field of pain treatment</u> in the following points:

3.1. Knowledge in pain-physiological mechanisms (pain memory, pain spiral, pain threshold, pain tolerance, ...)

☐	☐	☐	☐
sufficient	predominantly sufficient	predominantly insufficient	insufficient

3.2. Applying the WHO analgesic Ladder for pain management

☐	☐	☐	☐
sufficient	predominantly sufficient	predominantly insufficient	insufficient

3.3. Treating acute pain

☐	☐	☐	☐
sufficient	predominantly sufficient	predominantly insufficient	insufficient

3.4. Treating chronic pain

☐ sufficient ☐ predominantly sufficient ☐ predominantly insufficient ☐ insufficient

3.5. Treating psychological pain

☐ sufficient ☐ predominantly sufficient ☐ predominantly insufficient ☐ insufficient

3.6. Treatment with additional drugs (prevention of obstipation, antiemesis, ...)

☐ sufficient ☐ predominantly sufficient ☐ predominantly insufficient ☐ insufficient

3.7. Dealing with various forms of applications (oral, subcutaneous, transdermal, ...)

☐ sufficient ☐ predominantly sufficient ☐ predominantly insufficient ☐ insufficient

3.8. Application of complementary pain therapy methods (relaxation techniques, conversation, acupuncture, ...)

☐ sufficient ☐ predominantly sufficient ☐ predominantly insufficient ☐ insufficient

3.9. Treatment with additional drugs (antidepressants, anticonvulsants, corticosteroids, ...)

☐ sufficient ☐ predominantly sufficient ☐ predominantly insufficient ☐ insufficient

3.10. Dealing with pain scales

☐ sufficient ☐ predominantly sufficient ☐ predominantly insufficient ☐ insufficient

3.11. Documentation of pain

☐ sufficient ☐ predominantly sufficient ☐ predominantly insufficient ☐ insufficient

4. Please assess your occupational group's competence in the field of symptom control in the following points:

4.1. Gastrointestinal symptoms (nausea, emesis, singultus, obstipation, ileus)

☐ sufficient ☐ predominantly sufficient ☐ predominantly insufficient ☐ insufficient

4.2. Neurologic symptoms (vertigo, headaches, ...)

☐ sufficient ☐ predominantly sufficient ☐ predominantly insufficient ☐ insufficient

4.3. Pulmonal symptoms (pulmonary edema, dyspnea, , ...)

☐	☐	☐	☐
sufficient	predominantly sufficient	predominantly insufficient	insufficient

4.4. Urologic symptoms (dysuria, anuria, ...)

☐	☐	☐	☐
sufficient	predominantly sufficient	predominantly insufficient	insufficient

4.5. Dehydratation, cachexia

☐	☐	☐	☐
sufficient	predominantly sufficient	predominantly insufficient	insufficient

4.6. Psychological symptoms (angst, depression, confusion, ...)

☐	☐	☐	☐
sufficient	predominantly sufficient	predominantly insufficient	insufficient

4.7. Other:..

5. Did your academic education prepare you adequately for the care for critically ill and end-of-life patients?

☐	☐	☐	☐
yes	to a certain extent	predominantly not	noo

6. Please indicate whether your occupational group has a need for <u>qualified training opportunities or further training courses</u> on <u>pain therapy</u> by ticking the following boxes:

6.1. Pain-physiological mechanisms (pain memory, pain spiral, pain threshold, pain tolerance,...)

☐	☐	☐	☐
high	predominantly high	predominantly low	low

6.2. Dealing with pain scales

☐	☐	☐	☐
high	predominantly high	predominantly low	low

6.3. Applying the WHO analgesic Ladder for pain management

☐	☐	☐	☐
high	predominantly high	predominantly low	low

6.4. Treating acute pain

☐	☐	☐	☐
high	predominantly high	predominantly low	low

6.5. Treating chronic pain

☐	☐	☐	☐
high	predominantly high	predominantly low	low

6.6. Treatment with additional drugs (prevention of obstipation, antiemesis, ...)

☐ high ☐ predominantly high ☐ predominantly low ☐ low

6.7. Dealing with various forms of applications (oral, subcutaneous, transdermal, ...)

☐ high ☐ predominantly high ☐ predominantly low ☐ low

6.8. Application of complementary pain therapy methods (relaxation techniques, conversation, acupuncture, ...)

☐ high ☐ predominantly high ☐ predominantly low ☐ low

6.9. Treatment with additional drugs (antidepressants, anticonvulsants, corticosteroids, ...)

☐ high ☐ predominantly high ☐ predominantly low ☐ low

6.10. Documentation of pain

☐ high ☐ predominantly high ☐ predominantly low ☐ low

6.11. Other: ....

7. Please tell us whether your occupational group has a need for <u>qualified training opportunities</u> or further training courses on <u>symptom control</u> by ticking the following boxes:

7.1. Gastrointestinal symptoms (nausea, emesis, singultus, obstipation, ileus)

☐ high ☐ predominantly high ☐ predominantly low ☐ low

7.2. Neurologic symptoms (vertigo, headache, ...)

☐ high ☐ predominantly high ☐ predominantly low ☐ low

7.3. Pulmonal symptoms (pulmonary edema, dyspnea, , ...)

☐ high ☐ predominantly high ☐ predominantly low ☐ low

7.4. Urologic symptoms (dysuria, anuria, ...)

☐ high ☐ predominantly high ☐ predominantly low ☐ low

7.5. Dehydratation, cachexia

☐ high ☐ predominantly high ☐ predominantly low ☐ low

7.6. Psychological symptoms (angst, depression, confusion, ...)

☐ high ☐ predominantly high ☐ predominantly low ☐ low

7.7. Other: ....

8. Please tell us whether your occupational group has a need for **qualified training opportunities or further training courses** on **psychosocial care** for critically ill and end-of life patients and their relatives by ticking the following boxes:

8.1. Communication with patients

☐	☐	☐	☐
high	predominantly high	predominantly low	low

8.2. Communication with relatives

☐	☐	☐	☐
high	predominantly high	predominantly low	low

8.3. Handling of introductory conversations

☐	☐	☐	☐
high	predominantly high	predominantly low	low

8.4. Conducting difficult conversations (dying, death, mourning, “last things”, …)

☐	☐	☐	☐
high	predominantly high	predominantly low	low

8.5. Care of patients in their last days (terminal phase)

☐	☐	☐	☐
high	predominantly high	predominantly low	low

8.6. Care of relatives in the patient’s last days (terminal phase)

☐	☐	☐	☐
high	predominantly high	predominantly low	low

8.7. Care of relatives after the patient’s death

☐	☐	☐	☐
high	predominantly high	predominantly low	low

8.8. Ethical questions

☐	☐	☐	☐
high	predominantly high	predominantly low	low

8.9. Other: ....

9. Please tell us whether your occupational group has a need for <u>supporting/accompanying measures</u> to help them deal with <u>psychological strains</u> by ticking the following boxes:

9.1. Discussions with colleagues

☐	☐	☐	☐
high	predominantly high	predominantly low	low

9.2. Interdisciplinary discussions

☐	☐	☐	☐
high	predominantly high	predominantly low	low

9.3. Discussions right after a straining situation with a person of trust in a professional context

☐	☐	☐	☐
high	predominantly high	predominantly low	low

9.4. Supervision, balint groups

☐	☐	☐	☐
high	predominantly high	predominantly low	low

9.5. Seminars for dealing with critically ill and end-of-life patients

☐	☐	☐	☐
high	predominantly high	predominantly low	low

9.6. Other:**...**

10. Which of the following topics for qualified training opportunities would you rank the most important? Please indicate your priority order with numbers. (1: most important – 5: least important)

Ethical Questions	①	②	③	④	⑤
Symptom control	①	②	③	④	⑤
Your own strategies of coping	①	②	③	④	⑤
Pain therapy	①	②	③	④	⑤
Psycho-social care	①	②	③	④	⑤

11. At what time of the day to you prefer to take part in qualified training opportunities? Please indicate your preferable time of the day with numbers. (1: most convenient; 6: least convenient)

Evening classes /events	①	②	③	④	⑤	⑥
Afternoon classes /events	①	②	③	④	⑤	⑥
Day-time classes /events	①	②	③	④	⑤	⑥
Weekend classes /events	①	②	③	④	⑤	⑥
Compact courses (Monday to Friday)	①	②	③	④	⑤	⑥
Compact courses (Monday to Sunday)	①	②	③	④	⑤	⑥
Other:... ..	①	②	③	④	⑤	⑥

12. Would you bear the costs for qualified training opportunities or training courses?

☐ yes ☐ partly ☐ no (please go on with question 13)

If you ticked **"yes"** or **"partly":** What would be the maximum amount per year you would be willing to spend (in ÖS)?:.......................

13. Where would you attend qualified training opportunities? *(tick where applicable!)*

☐ In your area
☐ In the Steiermark
☐ In Austria
☐ Abroad

14. Which kind of set-ups do you prefer for qualified training opportunities or training in the following subject matters?

14.1. Ethical Questions	☐	interdisciplinary	☐	monodisciplinary
14.2 . Symptom control	☐	interdisciplinary	☐	monodisciplinary
14.3. Your own strategies of coping	☐	interdisciplinary	☐	monodisciplinary
14.4. Pain therapy	☐	interdisciplinary	☐	monodisciplinary
14.5. Psycho-social care/support	☐	interdisciplinary	☐	monodisciplinary
14.6. Other: …………………	☐	interdisciplinary	☐	monodisciplinary

15. How much time did you spend on training courses/ continued learning programs <u>in the last two years</u> which dealt with the following issues? Please indicate the time by writing down the <u>hours</u> you spent on those programs.

Palliative care /terminal care	... hrs
Symptom control	... hrs
Support of relatives of critically ill and end-of-life patients	... hrs
Pain therapy	... hrs
Psycho-social care/ support of critically ill or end-of-life patients	... hrs
Other:	... hrs

16. Please assess the importance of the cooperation among different occupational groups:

☐	☐	☐	☐
important	predominantly important	predominantly unimportant	unimportant

Thank you very much for your time!
