# Supplementary material for: Planning training seminars in palliative care: a cross-sectional survey on the preferences of general practitioners and nurses in Austria
Source: BMC Med Educ. 2010 Jun 11;10:43. doi: 10.1186/1472-6920-10-43 (PMC2893516; doi:10.1186/1472-6920-10-43)
Supplement: Additional file 2 — Questionnaire Nurses. Original questionnaire for the nurses (translation into English) [file 1472-6920-10-43-S2.PDF]

Questionnaire: mobile palliative care – nursing care

Graz, September 2000

Dear employees,

The care of critically ill or end-of-life patients has lately become an issue of growing interest. Internationally and in the Steiermark, hospitals are now planning to implement a graded care system for palliative patients.

The goal of the questionnaire is to learn more about the specific academic requirements and the need for accompanying measures of the occupational groups who work in nursing care or end-of-life care.

Your opinion is very important to us! That is why we would ask you to fill out the whole questionnaire. You will need approximately 15-20 minutes.
Please answer every question!

Your answers will be kept strictly confidential towards third parties. The results of this questionnaire will be online in spring 2001. In case you are interested, you will find the results on the website of the FAGW: http://www.stmk.gv.at/gesundheit/.

Thank you very much for your time and your cooperation!

Dir. Annemarie GIGL Dr. Johann BAUMGARTNER

Questionnaire
Academic requirements for mobile palliative care

In the following pages, you will find questions which will deal with your occupation. Please tick the boxes which apply to you (if not indicated otherwise). Please answer every question!

Personal information

Age: …… *years*

Sex: ☐ *male* ☐ f*emale*

Supporting organisation *(tick where applicable!)***:**

☐ *Caritas Hilfswerk Steiermark (relief organisation)*
☐ *Österreichisches Rotes Kreuz (Red Cross)*
☐ *Sozialmedizinischer Pflegedienst (nursing service)*
☐ *Volkshilfe Steiermark (public aid)*
☐ *Other:*

☐ **Self employed**

To which occupational group do you belong? *(please tick where applicable!)*

☐ DGKS/DGKP
☐ extended care
☐ caretakers /nurses for the elderly
☐ family caretaker

Work experience in the care system*: …… years*

1. Please assess your occupational group's competence concerning the psychosocial care for critically ill, end-of-life patients and their relatives in the following points:

1.1. Communication with patients

☐	☐	☐	☐
sufficient	predominantly sufficient	predominantly insufficient	insufficient

1.2. Communication with relatives

☐	☐	☐	☐
sufficient	predominantly sufficient	predominantly insufficient	insufficient

1.3. Handling of introductory conversations

☐ sufficient	☐ predominantly sufficient	☐ predominantly insufficient	☐ insufficient

1.4. Conducting difficult conversations (dying, death, mourning, “last things”, …)

☐ sufficient	☐ predominantly sufficient	☐ predominantly insufficient	☐ insufficient

1.5. Care of patients in their last days (terminal phase)

☐ sufficient	☐ predominantly sufficient	☐ predominantly insufficient	☐ insufficient

1.6. Care of relatives in the patient’s last days (terminal phase)

☐ sufficient	☐ predominantly sufficient	☐ predominantly insufficient	☐ insufficient

1.7. Care of relatives after the patient’s death

☐ sufficient	☐ predominantly sufficient	☐ predominantly insufficient	☐ insufficient

2. Please assess your occupational group’s competence in coping with emotional and physical strains in the following points:

2.1. Coping strategies for difficult situations

☐ sufficient	☐ predominantly sufficient	☐ predominantly insufficient	☐ insufficient

2.2. Coping with own pain

☐ sufficient	☐ predominantly sufficient	☐ predominantly insufficient	☐ insufficient

2.3. Coping with powerlessness

☐ sufficient	☐ predominantly sufficient	☐ predominantly insufficient	☐ insufficient

2.4. Coping with insecurities

☐ sufficient	☐ predominantly sufficient	☐ predominantly insufficient	☐ insufficient

2.5. Coping with guilty conscience

☐ sufficient ☐ predominantly sufficient ☐ predominantly insufficient ☐ insufficient

3. Please assess your occupational group's competence in the field of pain treatment in the following points:

3.1. Knowledge in pain physiological mechanisms (pain memory, pain spiral pain threshold, pain tolerance, ...)

☐ sufficient ☐ predominantly sufficient ☐ predominantly insufficient ☐ insufficient

3.2. Dealing with pain scales

☐ sufficient ☐ predominantly sufficient ☐ predominantly insufficient ☐ insufficient

3.3. Applying the WHO analgesic ladder for pain management

☐ sufficient ☐ predominantly sufficient ☐ predominantly insufficient ☐ insufficient

3.4. Treating acute pain

☐ sufficient ☐ predominantly sufficient ☐ predominantly insufficient ☐ insufficient

3.5. Treating chronic pain

☐ sufficient ☐ predominantly sufficient ☐ predominantly insufficient ☐ insufficient

3.6. Treating psychological pain

☐ sufficient ☐ predominantly sufficient ☐ predominantly insufficient ☐ insufficient

3.7. Treatment with additional drugs (prevention of obstipation, antiemesis, ...)

☐ sufficient ☐ predominantly sufficient ☐ predominantly insufficient ☐ insufficient

3.8. Documentation of pain

☐ sufficient ☐ predominantly sufficient ☐ predominantly insufficient ☐ insufficient

3.9. Dealing with various forms of applications (oral, subcutaneous, transdermal, ...)

☐	☐	☐	☐
sufficient	predominantly sufficient	predominantly insufficient	insufficient

3.10. Application of complementary pain therapy methods (relaxation techniques, conversation, acupuncture, ...)

☐	☐	☐	☐
sufficient	predominantly sufficient	predominantly insufficient	insufficient

3.11. Treatment with additional drugs (antidepressants, anticonvulsants, corticosteroids, ...)

☐	☐	☐	☐
sufficient	predominantly sufficient	predominantly insufficient	insufficient

4. Please assess your occupational group's competence in the field of <u>palliative care</u> in the following points:

4.1. Care of patients with gastrointestinal symptoms (nausea, emesis, singultus, obstipation, ileus)

☐	☐	☐	☐
high	predominantly high	predominantly low	low

4.2. Care of patients with neurologic symptoms (vertigo, headaches, ...)

☐	☐	☐	☐
high	predominantly high	predominantly low	low

4.3. Care of patients with psychological symptoms (angst, depression, confusion, ...)

☐	☐	☐	☐
high	predominantly high	predominantly low	low

4.4. Care of patients with pulmonal symptoms (pulmonary edema, dyspnea, , ...)

☐	☐	☐	☐
high	predominantly high	predominantly low	low

4.5. Care of patients with urologic symptoms (dysuria, anuria, ...)

☐	☐	☐	☐
high	predominantly high	predominantly low	low

4.6. Care of patients with dehydratation, cachexia

☐	☐	☐	☐
high	predominantly high	predominantly low	low

4.7. General and special patient bedding (decubitus prophylaxis, dyspnea, ...)

☐	☐	☐	☐
high	predominantly high	predominantly low	low

4.8. Special oral hygiene

☐ high ☐ predominantly high ☐ predominantly low ☐ low

4.9. Other:...

5. Did your education prepare you <u>adequately</u> for the treatment / care of critically ill and end-of-life patients?

☐ yes ☐ to a certain extent ☐ predominantly not ☐ not

6. Please indicate whether your occupational group has a need for <u>qualified training opportunities or further training courses</u> on <u>pain therapy</u> by ticking the following boxes:

6.1. Pain-physiological mechanisms (pain memory, pain spiral, pain threshold, pain tolerance,...)

☐ high ☐ predominantly high ☐ predominantly low ☐ low

6.2. Dealing with pain scales

☐ high ☐ predominantly high ☐ predominantly low ☐ low

6.3. Applying the WHO analgesic Ladder for pain management

☐ high ☐ predominantly high ☐ predominantly low ☐ low

6.4. Treating acute pain

☐ high ☐ predominantly high ☐ predominantly low ☐ low

6.5. Treating chronic pain

☐ high ☐ predominantly high ☐ predominantly low ☐ low

6.6. Treatment with additional drugs (prevention of obstipation, antiemesis, ...)

☐ high ☐ predominantly high ☐ predominantly low ☐ low

6.7. Documentation of pain

☐ high ☐ predominantly high ☐ predominantly low ☐ low

6.8. Dealing with various forms of applications (oral, subcutaneous, transdermal, ...)

☐ high ☐ predominantly high ☐ predominantly low ☐ low

6.9. Application of complementary pain therapy methods (relaxation techniques, conversation, acupuncture, ...)

☐ high ☐ predominantly high ☐ predominantly low ☐ low

6.10. Treatment with additional drugs (antidepressants, anticonvulsants, corticosteroids, ...)

☐ high ☐ predominantly high ☐ predominantly low ☐ low

6.11. Other: ....

7. Please tell us whether your occupational group has a need for qualified training opportunities or further training courses on <u>palliative care</u> by ticking the following boxes:

7.1. Care of patients with special oral hygiene

☐ high ☐ predominantly high ☐ predominantly low ☐ low

7.2. Care of cachectic patients (measures to stimulate appetite)

☐ high ☐ predominantly high ☐ predominantly low ☐ low

7.3. Care of patients with special needs in bedding (decubitus prophylaxis, dyspnea, ...)

☐ high ☐ predominantly high ☐ predominantly low ☐ low

7.4. Care of patients with nausea or emesis

☐ high ☐ predominantly high ☐ predominantly low ☐ low

7.5. Care of patients with neuro-psychological symptoms

☐ high ☐ predominantly high ☐ predominantly low ☐ low

7.6. Other: ....

8. Please tell us whether your occupational group has a need for <u>qualified training opportunities or further training courses</u> on <u>psychosocial care for critically ill and end-of life patients and their relatives</u> by ticking the following boxes:

8.1. Communications with patients

☐ high ☐ predominantly high ☐ predominantly low ☐ low

8.2. Communications with relatives

☐ high ☐ predominantly high ☐ predominantly low ☐ low

8.3. Communications on coping issues

☐ high ☐ predominantly high ☐ predominantly low ☐ low

8.4. Conducting difficult conversations (dying, death, mourning, "last things", …)

☐ high ☐ predominantly high ☐ predominantly low ☐ low

8.5. Care for patients in their last days (terminal phase)

☐ high ☐ predominantly high ☐ predominantly low ☐ low

8.6. Care for relatives in the patient's last days (terminal phase)

☐ high ☐ predominantly high ☐ predominantly low ☐ low

8.7. Care for relatives after the patient's death

☐ high ☐ predominantly high ☐ predominantly low ☐ low

8.8. Ethical questions

☐ high ☐ predominantly high ☐ predominantly low ☐ low

8.9. Other: ....

9. Please tell us whether your occupational group has a need for <u>supporting measures</u> to help them deal <u>with psychological strains</u> by ticking the following boxes:

9.1. Discussions with colleagues

☐ high ☐ predominantly high ☐ predominantly low ☐ low

9.2. Interdisciplinary discussions

☐ high ☐ predominantly high ☐ predominantly low ☐ low

9.3. Discussions right after a straining situation with a person of trust in a professional context

☐ high ☐ predominantly high ☐ predominantly low ☐ low

9.4. Supervision, balint groups

☐ high ☐ predominantly high ☐ predominantly low ☐ low

9.5. Seminars on dealing with critically ill patients and end-of-life care

☐ high ☐ predominantly high ☐ predominantly low ☐ low

9.6. Other :...

10. Which of the following topics for <u>qualified training opportunities</u> would you rank the most important? Please indicate your <u>priority</u> order with numbers. (1: most important – 5: least important)

Ethical Questions	①	②	③	④	⑤
Pain therapy	①	②	③	④	⑤
Palliative care	①	②	③	④	⑤
Your own strategies of coping	①	②	③	④	⑤
Psychosocial care	①	②	③	④	⑤

11. At what <u>time</u> of the day to you prefer to take part in <u>qualified training opportunities</u>?
Please indicate your preferable time of the day with numbers. (1: most convenient; 6: least convenient)

Evening classes /events	①	②	③	④	⑤	⑥
Afternoon classes /events	①	②	③	④	⑤	⑥
Day-time classes /events	①	②	③	④	⑤	⑥
Weekend classes /events	①	②	③	④	⑤	⑥
Compact courses (Monday to Friday)	①	②	③	④	⑤	⑥
Compact courses (Monday to Sunday)	①	②	③	④	⑤	⑥
Other:..	①	②	③	④	⑤	⑥

12. Would you bear the costs for qualified training opportunities or training courses?

☐	☐	☐
yes	predominantly	no

If you ticked **"yes**" or "**predominantly":** What would be the maximum amount per year you would be willing to spend (in ÖS)?: ....

13. Would you take part in qualified training opportunities in your leisure time?

☐	☐	☐	☐
yes	sometimes	rarely	not at all

14. Where would you attend qualified training opportunities?
(tick where applicable!)

☐ In your area
☐ In the Steiermark
☐ In Austria
☐ Abroad

15. Which kind of set-ups do you prefer for qualified training opportunities or training in the following subject matters?

15.1. Pain therapy	☐	interdisciplinary	☐	monodisciplinary
15.2. Palliative care	☐	interdisciplinary	☐	monodisciplinary
15.3 Psycho-social care	☐	interdisciplinary	☐	monodisciplinary
15.4. Your own strategies of coping	☐	interdisciplinary	☐	monodisciplinary
14.5. Ethical questions	☐	interdisciplinary	☐	monodisciplinary

16. How much time did you spend on training courses/ continued learning programs <u>in the last two years</u> which dealt with the following issues? Please indicate the time by writing down the hours you spent on those programs.

	Hours
End-of-life care	...
Palliative care	...
Support of relatives of critically ill and end-of-life patients	...
Pain therapy	...
Psycho-social care/ support	…

17. Please assess the importance of the cooperation among different occupational groups:

☐	☐	☐	☐
important	predominantly important	predominantly unimportant	unimportant

Thank you very much for your time!
